# Helping Reasoners Succeed in the Wason Selection Task: When Executive Learning Discourages Heuristic Response but Does Not Necessarily Encourage Logic

**DOI:** 10.1371/journal.pone.0123024

**Published:** 2015-04-07

**Authors:** Sandrine Rossi, Mathieu Cassotti, Sylvain Moutier, Nicolas Delcroix, Olivier Houdé

**Affiliations:** 1 Laboratory for the Psychology of Child Development and Education, Sorbonne, CNRS Unit 8240, Paris, France; 2 Laboratoire de Psychopathologie et Processus de Santé, EA 4057, Paris, France; 3 CNRS, UMS 3408, GIP CYCERON, Caen, France; 4 University of Caen Low Normandy, Caen, France; 5 Normandy Alliance for Higher Education and Research, Caen University, Caen, France; 6 Paris Descartes University, Paris, France; 7 Sorbonne-Paris-Cité Alliance for Higher Education and Research, Paris Descartes University, Paris, France; 8 Institut Universitaire de France, Paris, France; Universiteit Utrecht, NETHERLANDS

## Abstract

Reasoners make systematic logical errors by giving heuristic responses that reflect deviations from the logical norm. Influential studies have suggested first that our reasoning is often biased because we minimize cognitive effort to surpass a cognitive conflict between heuristic response from system 1 and analytic response from system 2 thinking. Additionally, cognitive control processes might be necessary to inhibit system 1 responses to activate a system 2 response. Previous studies have shown a significant effect of executive learning (EL) on adults who have transferred knowledge acquired on the Wason selection task (WST) to another isomorphic task, the rule falsification task (RFT). The original paradigm consisted of teaching participants to inhibit a classical matching heuristic that sufficed the first problem and led to significant EL transfer on the second problem. Interestingly, the reasoning tasks differed in inhibiting-heuristic metacognitive cost. Success on the WST requires half-suppression of the matching elements. In contrast, the RFT necessitates a global rejection of the matching elements for a correct answer. Therefore, metacognitive learning difficulty most likely differs depending on whether one uses the first or second task during the learning phase. We aimed to investigate this difficulty and various matching-bias inhibition effects in a new (reversed) paradigm. In this case, the transfer effect from the RFT to the WST could be more difficult because the reasoner learns to reject all matching elements in the first task. We observed that the EL leads to a significant reduction in matching selections on the WST without increasing logical performances. Interestingly, the acquired metacognitive knowledge was too “strictly” transferred and discouraged matching rather than encouraging logic. This finding underlines the complexity of learning transfer and adds new evidence to the pedagogy of reasoning.

## Introduction

Reasoning skills are a core component of intelligence [1]. Unfortunately, reasoning is subject to cognitive biases. Reasoners make systematic logical errors by giving heuristic responses that reflect deviations from logical norms. Dual process theories of reasoning explain this human tendency to violate the normative standards of logic by distinguishing two cognitive systems [2]-[3]. System 1 is defined as a set of autonomous sub-systems that reflect the domain-specific nature of learning, which encompasses rapid, parallel, automatic, and effortless processes (heuristic) by permitting conscious access only to the final production. System 2 is necessary for hypothetical thinking, and its (analytic) processes are slow, sequential, controlled, effortful, and require the working memory system and specifically executive processing. Dual process theories propose that the two systems often interact. Fast and unconscious processes based on prior knowledge and beliefs may provide correct responses but may also lead to bias in situations that require more complex reasoning. In these cases, the analytic system must override the belief-based responses produced by the heuristic system [4].

The ability to avoid these heuristics and biases is thought to be related to cognitive control processes necessary to inhibit the response given by system 1 to activate responses from system 2 thinking [5]. Inhibition of irrelevant information or irrelevant strategies is at the core of the human cognitive system and its development [6–8]. The emphasis is on executive functions, which refer to high-level cognitive control processes that underlie goal-directed behavior. Inhibition processes are not only capable of resisting distraction but also of avoiding the eruption of routine behavior schemas that are ineffective, particularly in unfamiliar situations or when new problems must be solved. Houdé and Moutier [9] developed the original executive learning approach that seeks to redirect reasoners’ thinking. The aim of this approach is to lead reasoners to inhibit heuristic responses by introducing, in the learning instructions, several elements in the form of verbal and visuo-spatial executive warnings of the error risk (i.e., a “hot” type of learning). The pre- and post-learning effects were tested in another reasoning task that is known to activate the same erroneous heuristic response.

**Fig 1:**
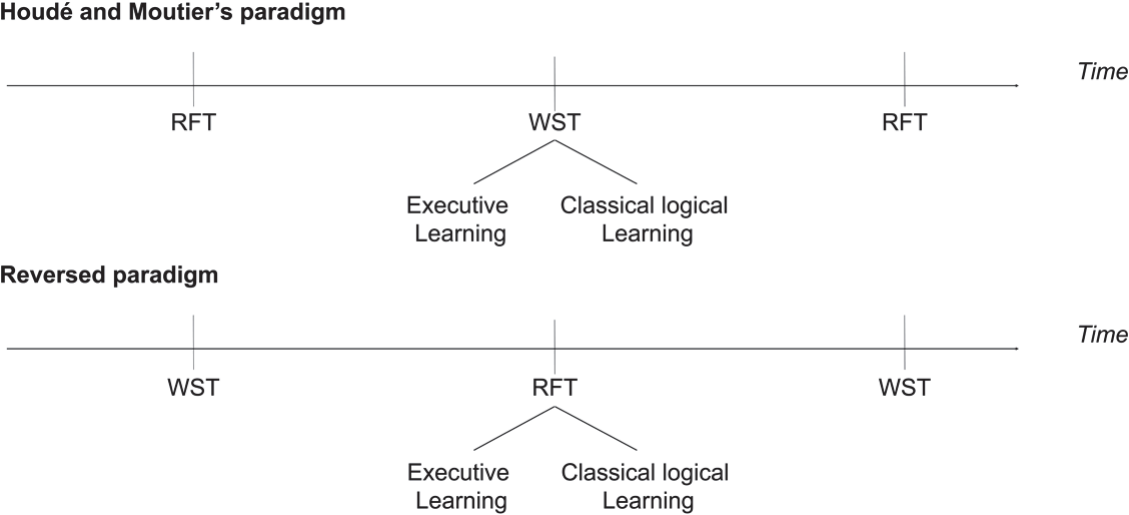
The study design used by Houdé and Moutier [9] and the design of our reversed paradigm.

Executive learning (EL) induced significant metacognitive transfer in child and adult reasoners who exhibited fewer heuristic responses after EL than after to classical logical learning [9–12]. Metacognitive experiences are understood to encompass all conscious cognitive or affective experiences associated with the solving of a particular problem [13]. These transient experiences occur especially when the participant is performing a complex cognitive task that requires the implementation of control processes. However, there is an important remaining issue for the domain. It is not known whether the metacognitive transfer of knowledge acquired with the EL is always effective when the order of the learning task and the pre/post task are reversed. The present study answers this question directly by testing adults with the reverse of Houdé and Moutier’s original reasoning paradigm (Fig 1).

A famous reasoning task is the Wason selection task (WST). It includes four cards, each of which is presented as having a letter a letter on one side and a number on the other side. Two of the cards have a letter on their exposed side, and two have a digit on their exposed sides. These cards read A, D, 3, and 7. These cards are also associated with the following conditional rule: if there is an A on one side of the card, then there is a 3 on the other side of the card. Logically, the first part of a conditional rule is called the antecedent, and the second part is called the consequent. The participant is told that this rule applies to the four cards shown and may be true or false. Further, he/she is asked to choose which card to turn over to decide whether the rule is true or false. Logically, the A card represents an affirmation of the antecedent (true antecedent), the D card represents a falsification of the antecedent (false antecedent), the 3 card represents an affirmation of the consequent (true consequent), and the 7 card represents a falsification of the consequent (false consequent). Logic requires that the person chooses to turn the A and 7 cards over (true antecedent and false consequent) because only this combination can falsify the rule and prove whether it is true or false (i.e., finding a card that has an A on one side and does not have a 3 on the other side, or finding a card that does not have a 3 on one side (here the 7 card) and has an A on the other side would falsify the rule). Despite the apparent simplicity of this popular reasoning problem [14], few participants give the correct answer, and most choose to turn the A and 3 cards or the A card only, which match the cards described in the conditional rule.

Many interpretations have been proposed to account for the difficulty encountered by the reasoners in the WST (see [15] for a review). A first attempt was to suppose that reasoners choose only those cards that would confirm the given conditional rule rather than refute it. This is the confirmation bias [16]. Another possible way to interpret performance of the WST refers to conversational maxims by Grice [17]. People may understand the presented conditional rule as biconditional (If *and only if* there is an A then there is a 3). Cheng and Holyoak [18] proposed that people often reason using pragmatic reasoning schemas. These rules are highly generalized and abstracted but nonetheless defined with respect to classes of goals and types of relationships such as the “permission” situations which improve performance on WST (If one is to take action A then one must satisfy precondition P). Cheng et al. [19] observed a learning effect on the WST using conditional rule training added to an example training based on pragmatic versions of the WST. However, these pragmatic versions lead reasoners to check the violation rather than the truth of the conditional rule as suggested in the framework of evolutionary psychology [20]. We know that reasoners performed successfully when they have to detect cheaters in a social contract. Several researchers [21]-[22] have noted that the WST is not a mere logical reasoning task. Therefore, the task may have two aspects: interpretation of the conditionals and decision making in regard to which cards to select.

More recently, the WST has been acknowledged as a task that enables us to explore dual processes of thinking [23]. In the WST, System 1 processes compete with System 2 processes in determining the choice of cards. Heuristic processes are rapid, parallel, automatic, and effortless. They involve the allocation of selective attention towards the matching cases A and 3 (true antecedent and true consequent) and deem the other irrelevant [24]. This allocation implies triggering the intuitive response that is not logical (chose the A and 3 cards). The implication of these heuristic processes in the WST has been corroborated by Roberts and Newton [25]. Although authors found that reasoners’ responses in a rapid response time task did not radically differ from responses given in the free response time version, they also found an increase in the selection of the matching 3 card in the rapid response time task. The rapid response time condition permitted the activation of System 1 processes and the free response time condition did not permit the activation of System 2 processes. Reorienting reasoners’ knowledge using questionnaires is a way of constraining the possible range of misinterpretations, and it guides them to a logical interpretation in which they can reason correctly [26]-[27]. Osman [28] combined a tutoring procedure based on identifying and modifying the misinterpretations reasoners had of the task [29] and time constraints. The tutoring was based on the same version of WST that was used in pre- and post-test. She observed that this tutoring is effective when available cognitive resources are reduced using rapid response or rapid presentation task, opposed to a condition without tutoring. System 1 can no longer trap them even when they are placed in a rapid response time condition.

To solve the WST, the reasoner requires hypothetical thinking to select cards A and 7 (true antecedent and false consequent) that only System 2 could provide. Therefore, there would be a conflict between heuristic and analytic output. This conflict-monitoring in dual reasoning processes has recently been examined by De Neys and collaborators [30–32]. These authors showed that people detect the conflict between the output of the two systems and argued that reasoning errors occur not because people failed to monitor System 1 but because they failed to inhibit the pre-attentional response given by System 1 (for a review see [5]). The present article focuses on the assumption that people without gaps in normative logic could still be poor inhibitors [33]. This idea is consistent with several studies that support the contribution of executive processing capacity to reasoning at the behavioral level both in adults [34–36] and children [37–40].

Houdé and Moutier [9] developed the EL for teaching adult participants to inhibit the matching heuristic response primed by System 1 to activate the logical response provided by System 2 in the WST. These authors introduced warning elements in the form of verbal and visuo-spatial executive warnings with the aim of teaching subjects how to inhibit matching heuristic strategies and also provided the logical explanation underlying the task to activate the analytic strategy. The experimental training began with, "In this problem, the source of the error lies in a habit we all have of concentrating on cards with the letter or number mentioned in the rule (the experimenter points to cards A and 3 and to the place where they are mentioned in the rule) and not paying attention to the other cards. This factor can have a very misleading effect on us: we think this makes things easier when in fact we're falling into a trap!" Then, for each matching response (A, A-3) printed on a card, the subject was led to explain why it is misleading and then to put it under a transparent hatched area, which depicted inhibition. According to Perner [41], inhibition and set shifting (or cognitive flexibility) depend on a metarepresentation of the habitual act as maladaptive. These authors chose to subtract the executive warnings from the learning procedure and therefore retain the logical components as a classical logical learning condition (CL).

To determine whether EL or CL were sufficient to improve performance, Houdé and Moutier [9] presented the rule falsification task (RFT; [42]) to participants in a pre-test. Performance of this task required the same inhibition of a matching heuristic strategy at the attentional level and access to the same analytic strategy as the WST presented above (this factor is an essential feature of the design). The participants were asked to select two colored shapes (among circles, diamonds, and squares in blue, yellow, red, and green) to falsify the following conditional rule with which they were presented: if there is not a red square on the left, then there is a yellow circle on the right. Similar to the WST, few participants gave the logical answer by selecting, on the left, a shape that was not a red square (true antecedent) and selecting, on the right, a shape that was not a yellow circle (false consequent). Most participants selected the red square and the yellow circle (false antecedent and true consequent) (i.e., the shapes that matched those described in the rule). After the pre-test, participants were selected based on their poor performance on the RFT. Then, half of the unsuccessful participants received EL, and the others received CL. Instead of being limited to practice or instruction about the pre-learning task, the learning aimed to help subjects transfer a self-regulatory—or self-feeling—mechanism (also called autoregulation) from the WST to the RFT. Both types of learning were based on the WST, which is a formally identical reasoning task.

The summary of the responses on the WST at the beginning of the training phase observed in previous studies [9], [12] indicated massive failure. There was not a single correct answer (A-7) in the entire sample. The most frequent wrong answer, observed in half the participants, was A-3 that corresponded to the matching bias. The answers A or 3, which are called partial matching, were given by 28% of the participants (22% and 6%, respectively). The remaining 22% of the participants gave various incorrect answers including 7, A-D-7, A-D, and D-7. Therefore, a matching strategy (A-3, A, or 3) appears to be the typical bias triggered by WST; 78% of the sample relied on total or partial matching. In Moutier, Angeard, and Houdé’s study [12], the pre-test results indicated overall that participants’ logical performance in both the EL and CL conditions was very low (by construction, the scores of the two groups were comparable). The participants’ scores were divided them into two sub-populations: a biased group (n = 15) and a logical group (n = 3). The clearly predominant group exhibited biased behavior, which in the present case corresponded to the matching bias (total or partial: matching with the figure mentioned in the antecedent and/or consequent: red square and/or yellow circle). What were the respective proportions of the biased and logical sub-populations within the EL condition compared to the CL condition, on the post-test? The results showed that the proportions changed substancially between the pre- and post-tests. After EL, the sample could no longer be called globally biased. However, the CL sample, which had undergone no inhibition-component training (in which, by subtraction, only the logical components remained), had a clearly predominant matching-bias sub-population. An intra-individual analysis of changes from biased to logical behavior between the pre-test and the post-test indicated a training effect (existence of biased logical patterns) in the EL condition only (no effect in the CL condition). This finding indicates that executive warnings alone were helpful in managing to inhibiting the tempting matching heuristic generated by System 1.

The significant effect of EL in managing the inhibition of matching heuristic in conditional reasoning has been corroborated in a brain imaging study of adults [43]. Data provided evidence of a striking shift from the posterior part of the brain (a perceptual network that reflects the matching heuristic) when subjects were erroneous in the pre-test to a prefrontal network when they performed correctly in the post-test that was present only at the time at which inhibitory control was occurring. Note that this neuroanatomical shift was not observed after CL that was based on only the logical component of the task. The contribution of executive processes in overcoming the matching heuristic at the neural level has also been corroborated [44]-[45]. Furthermore, after EL (at the moment when the error-to-logic shift occurred), greater cerebral activity was observed in the right ventromedial prefrontal cortex [46]. This region is known to be implicated in the processing of risk, fear, and decision-making and is engaged in the recovery of previous emotional experiences to guide strategy selection [47]. The recruitment of this area can be interpreted as the retrieval of the emotional experience triggered by the executive warnings contained in the learning phase. Cassotti and Moutier [10] have recently showed that the emotional component of these warnings is essential for receptivity to this learning.

The behavioral and neural data provide evidence implicating inhibitory processes and open the doors to a neuropedagogy of reasoning [48]-[49]. Considering the efficiency with which EL has been observed to redirect reasoners’ thinking, investigating the generalization of EL is essential. Interestingly, the tasks used by Houdé and Moutier [9] differed in presentation and also in the metacognitive cost of inhibiting the heuristic (Table 1). The WST includes no negation in the conditional (if there is an A on one side of the card, then there is a 3 on the other side of the card) and requires half-suppression of the matching elements (the 3 card) for a correct answer. In contrast, the RFT includes a negation in the conditional (if there is not a red square on the left, then there is a yellow circle on the right) and necessitates a simple global rejection of the matching elements for a correct answer. Therefore, depending on whether one uses the WST or the RFT during the learning phase, metacognitive learning difficulty is expected to differ. We investigated this difficulty and various matching-bias inhibition effects in a new (reversed) paradigm.

**Table 1:** For each reasoning task, we present the analysis of the conditional rule, the classical erroneous response that matches with the elements presented in the conditional rule, and the expected logical response that partially matches (WST) or not (RFT) with these elements.

	True antecedent	False antecedent	True consequent	False consequent	Erroneous response / Matching cases	Correct response
**WST**	A	Not A (D)	3	Not 3 (7)	A and 3	A and not 3
**RFT**	Not a red square	Red square	Yellow circle	Not a yellow circle	Red square and yellow circle	Not a red square and not a yellow circle

The participants were presented with the WST in pre- and post-tests and taught to inhibit the matching heuristic in the RFT (Fig 1). Because the two reasoning tasks are formally equal, one could hypothesize a positive effect of EL. Alternatively this effect could be modulated according to the reasoning task to which EL is applied. When the learning applies to the WST, as in the original paradigm, the inhibition of only one matching case named in the rule was taught; however, when the learning applies to the RFT, as is the case in the current study, inhibition of all matching cases named in the rule is taught because the rule includes a negation in the antecedent. In this condition, the metacognitive transfer of EL may be more “strict” because in the RFT, the experimenter learns to inhibit all matching cases to succeed. Then, reasoners may inhibit all matching cases in the WST presented in the post-test, although the logical answer is associated with more fine-grained partial inhibition of only one matching case.

## Material and Methods

### Participants

Sixty French undergraduate students from the University of Caen (France) participated in this experiment. The present study was conducted in accordance with the Declaration of Helsinki and was approved by the local ethic committee of the LaPsyDE (CNRS, and Universities of Paris Descartes and Caen). All participants (or their parent or guardian) provided written informed consent. No reward was offered. They were unaware of the psychology of reasoning principles. The same experimenter tested individuals separately, and testing occured in two sessions held one day apart. In the pre-test, the participants were subjected to the WST. We observed that 42 participants failed the task, and 18 participants succeeded, wich was consistent with the literature on the WST. We excluded participants who had succeeded in the WST because there was no reason to teach them how to solve the task. Most participants who had failed the WST exhibited erroneous matching responses (total, A-3, or partial, A or 3, n = 30) and others who gave erroneous responses without exhibiting the matching strategy (n = 12). Consistent with the results reported in the literature, the participants clearly exhibited biased behavior in favor of a matching heuristic strategy (binomial test significant at. 002, with n = 42 and k = 12; see [50]). The sample was divided into an executive learning group (EL, n = 21, mean age 20.12 ± 3.35 years; 17 women) and a classical logical learning group (CL, n = 21, mean age 19.84 ± 1.11 years; 18 women) based on their responses to the WST. These groups were comparable in terms of WST failure distribution (number of erroneous matching responses, A-3, A or 3, and erroneous responses without exhibiting the matching strategy; χ_(2)_^2^ = 1.19, *p* = .40) and age (t_(40)_ = -0.13, *p* = .89). The EL group received instructions to inhibit the matching heuristic with verbal and visuo-spatial executive warnings in addition to the instructions on the logic of conditional reasoning, whereas the CL group received the same instructions on the logic of conditional reasoning without the executive components. The second session consisted of learning the RFT (we alternately tested the participants in the two types of learning, executive or control) and a post-test in which the participants were tested again on the WST.

### Design and procedure

**Fig 2:**
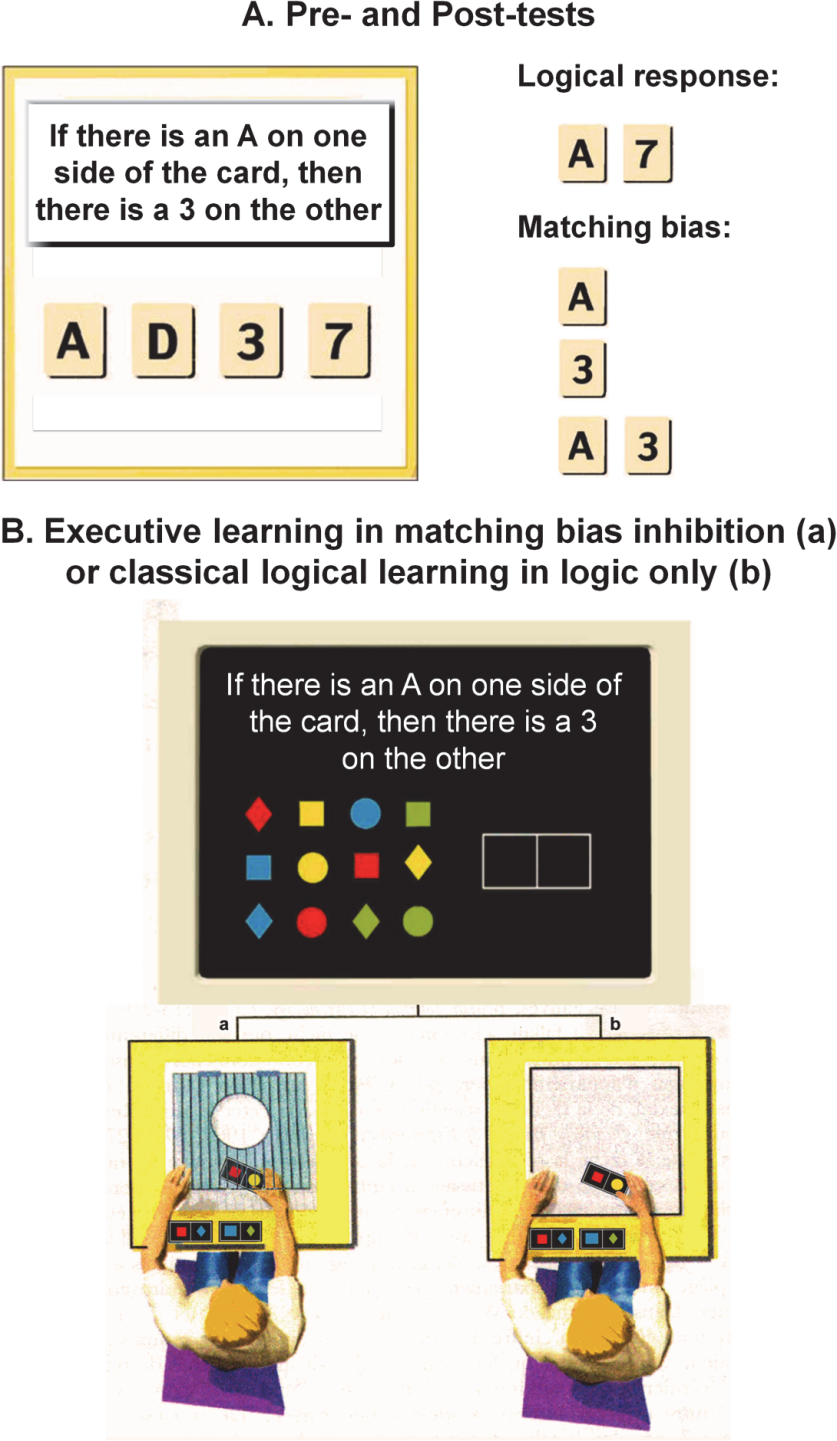
Materials used in the pre-test, the learning phase, and the post-test. A—English translation of the pre- and post-tests materials used for the four-card selection task. B—Materials used in the learning stages of the conditional rule falsification task: (a) the board depicts the executive processes (inhibition, shown as hatching) required for correct task performance during EEL; (b) classical logical learning without the inhibition component (only the logical explanation of the incorrect and the correct answers).

In the pre-test, the participants were presented with the conditional rule “*if there is an A on one side of the card*, *then there is a 3 on the other side of the card”* and four cards, *A*, *D*, *3*, *7*, each of which was known to have a letter on one side and a number on the other. The experimenter sat near the participant, a position held constant across the participants. The cards were lined up on a board with the rule written across the top (Fig 2A). The task was to decide which card(s) needed to be turned over to determine whether the rule was true or false.

The RFT was used to support learning and lasted approximately 30 to 45 min. The participants were presented with the conditional rule “*if there is not a red square on the left*, *then there is a yellow circle on the right*” and twelve colored geometric figures (*squares*, *circles*, *and diamonds in green*, *red*, *yellow and blue*; see Fig 2B). The students had to falsify the conditional rule by putting two colored figures, one for the antecedent and the other for the consequent, in a double box drawn on the board. Afterwards, they were subjected to EL with executive warnings or CL with logical components only. The learning always began upon the participants’ responses.

EL consisted of presenting the participants with an analysis of the task and the trap the task involved. EL explicitly imbued its warning elements as shown in italics in the following instructions:

“In this problem, the source of the error lies in a habit we all have of concentrating on geometric figures with the shape or color mentioned in the rule [here, the experimenter points to the red square and the yellow circle and to the place they were mentioned in the rule] and not paying attention to the other figures. This can have a very misleading effect on us: we think this makes things easier *when in fact we’re falling into a trap*! Thus, the goal here is *(1) to not fall into the trap of the two colored figures*, *the red square and the yellow circle mentioned in the rule and (2)* to consider all of the colored figures, squares, circles, diamonds in green, red, yellow and blue [here, the experimenter points to all the figures] by imagining if each one can or cannot be placed on the left or the right of the double box drawn on the board [here, the experimenter points the double box] to see whether these figures can make the rule false. To help you understand, let’s consider the different answers and eliminate the wrong ones—*the ones that make you fall into the trap*—to find the right answer”.

Next, the participant was shown the second part of the experimental materials, which consisted of another board placed next to the first on which the response repertoire was depicted as a box, the inhibition process as a transparent hatched area on top of the box, and the activation process was depicted as an unhatched circle cut-out from the middle of the hatched area (see Fig 2B, board a). Some answers were represented on small cards that could be slid into the response repertoire. These answers were different colors that depended on whether the answer was correct (green) or incorrect (red). The student was invited to use this material to learn to avoid falling into the trap. If he/she had trouble understanding and could not explain the trap or how to avoid it to succeed, the experimenter helped him/her by repeating the entire explanation. The learning began with the study of the participant’s responses on the RFT and ended when he/she was capable of producing correct explanations (without prompting from the experimenter) for the incorrect answers. The CL (Fig 2B, board b) was identical to the procedure for the EL described above except that it lacked the executive warnings (both verbal and visuo-spatial). The participants were read the same instructions as those in the EL, except the sentences that are italicized. Therefore, the remaining learning instructions contained the logical explanation of the correct and incorrect answers. The complete instructions used in this study are presented in (see S1 Dataset). The post-test was administered immediately after learning and involved the participant being tested again with the WST.

## Results

Results are presented in two sections. First, we present the performance observed on the rule falsification task (RFT) before the learning stage, and second, the performances observed on the Wason selection task (WST), which measured the transfer effect from the pre-test to the post-test. For each task we computed the participant’s responses in terms of matching (total or partial) or logical responses. To capture the fine-grained transfer effect on the WST for the two learning groups (EL and CL), we computed the selection (1) or the non-selection (0) of each card (A, D, 3 and 7). We used non-parametrical and parametrical statistical tests to test the effects of the Test (pre *vs*. post) and the type of Learning (executive *vs*. logical) factors. Raw values are presented in (see S1 Dataset).

### Performance on the learning task (RFT)

The participants were subjected to the RFT before the learning stage. Most gave incorrect responses (39 out of 42), and the most frequent incorrect answer corresponded to classical matching (34 out of 39), which occurs when the paticipants matched the elements in their answers exactly (red square and yellow circle). Partial matching (5 of 39) was defined based on the same principle but was limited to a single item, the antecedent (red square and not a yellow circle) or the consequent (not a red square and yellow circle). Finally, only three participants gave the logical answer, and one of these exhibited reversed matching by taking the two items named in the rule and switching their location (yellow circle and red square). The response distributions were similar for both the EL and CL groups (χ_(1)_^2^ = .36, *p* = .55 for correct *versus* incorrect answers and χ_(2)_^2^ = 2.25, *p* = .32 for total matching, partial matching and logical response).

### Transfer effects in the post-test (WST)

**Fig 3:**
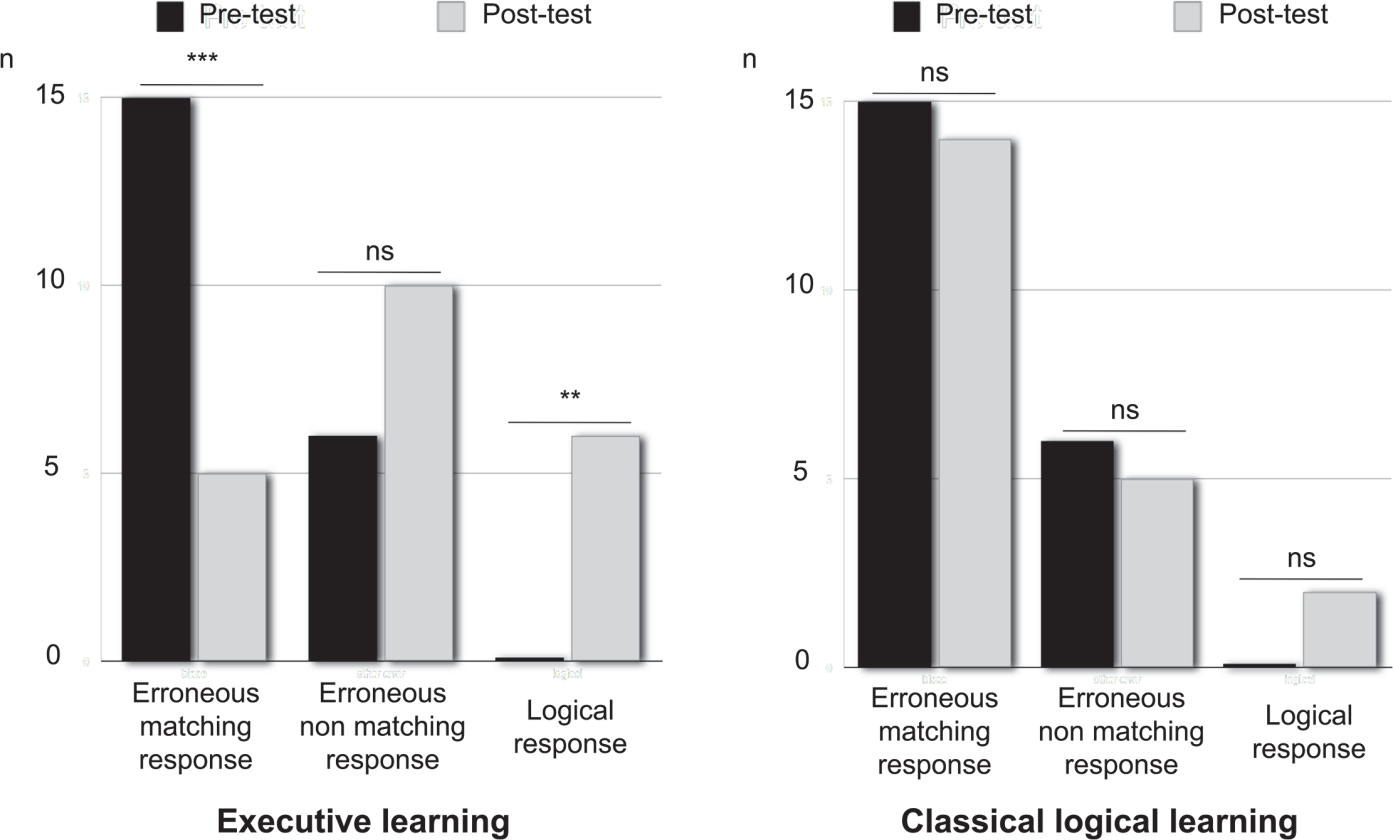
Response distribution for the four-card selection task. The results are shown for the pre-test before the learning stage and for the post-test after EEL or classical logical learning.

As Fig 3 shows, we observed a significant difference in the response distributions between the two groups that depended on the learning they had previously received (χ_(2)_^2^ = 7.92, *p* = .02). The response distribution observed after EL indicated that these participants could no longer be called biased according to the matching heuristic in the WST (χ_(2)_^2^ = 2.47, *p* = .11). The observed percentage of logical responses was higher in the post-test (26.6% logical responses and 71.4% erroneous matching and non-matching responses) than in the pre-test, in which none of the participants gave the logical response (*p* = .02, Fisher’s exact test). Furthermore, although this learning group exhibited behavior that was biased in favor of a matching heuristic strategy in the pre-test (71.4% erroneous matching responses and 28.6% erroneous non-matching responses), this pattern was reversed (33.3% *vs*. 66.7%, respectively, *p* = .04, Fisher’s exact test) in the post-test. The number of individual response patterns observed in the post-test for both the EL and CL groups is presented in the Table 2.

**Table 2:** Number of individual response patterns observed in the post-test for both the executive learning (EL) and classical logical learning (CL) groups.

	EL	CL
**Logical answer**		
TA, FC	6	2
**Biased answer**		
Total matching TA, TC	0	9
Partial matching TA	5	5
**Other error**		
FA, FC	4	1
TC, FC	0	1
TA, TC, FC	2	0
FA, TC, FC	2	1
TA, FA, TC, FC	2	2
	**21**	**21**

TA = true antecedent; FA = false antecedent; TC = true consequent; FC = false consequent; respectively A, D, 3, 7.

The CL group, who received no executive warnings during learning (i.e., by subtraction, only the logical components remained), was predominantly biased in their performance of the WST (χ_(2)_^2^ = 11.14, *p* = .003). The percentage of logical responses was not significantly different between the post-test (9.5% logical responses and 90.5% erroneous matching and non-matching responses) and the pre-test, in which none of the participants gave the logical response (*p* = .49, Fisher’s exact test). Furthermore, this learning group exhibited behavior biased in favor of a matching heuristic strategy in the post-test (73.7% erroneous matching responses and 26.3% erroneous non-matching responses) compared to the pre-test (71.4% *vs*. 28.6%, respectively, *p* = 1, Fisher’s exact test).

**Fig 4:**
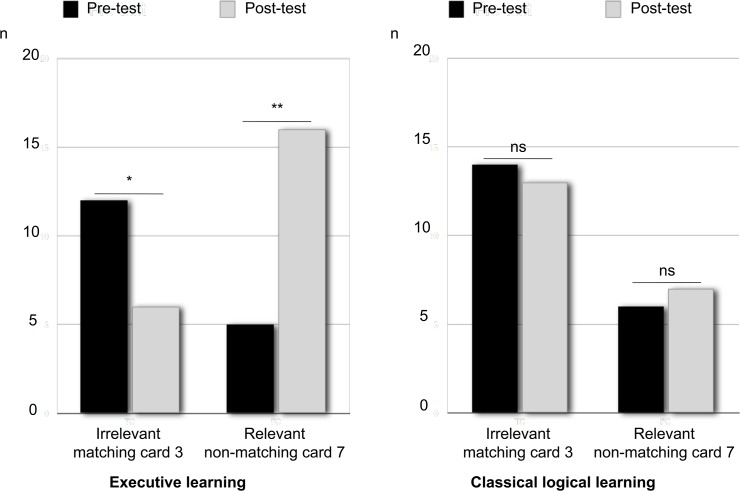
The numbers of the card selected in the pre- and post-tests after EL or classical logical learning. Here, we report the results of the matching card (3, the true consequent) and the non-matching card (7, the false consequent).

Next, we conducted intra-individual analyses of the behavioral changes between the pre- and post-tests to capture the fined-grained learning effects in the two learning groups. We studied the selection of the critical cards to access the logical response in the WST: the 3 card, which is the true consequent that the participant should inhibit after learning in favor of the selection of the 7 card, the false consequent. We ran an Analysis of Variance (with a generalized linear model, see [51]) with Test (pre/post) and Learning (executive/control) as independent factors. We observed a significant effect of Learning on the selection of the 3 card (*F*_(1, 40)_ = 3.9; *p* = .04, η^2^_p_ = .002). The 3 card was selected less frequently by the participants who received EL. Interestingly, as shown in Fig 4, we observed a significant interaction between Test and Learning for selection of the 7 card (*F*_(1, 40)_ = 6.71; *p* = .01, η^2^_p_ = .14). The planned comparisons revealed that the frequency of selection of the 7 card was equal between the EL and the CL groups in the pre-test (*F*_(1, 40)_ = 3.22; *p* = .08) and between the pre-test and the post-test in the CL group (*F*_(1, 40)_ = 0.13; *p* = .72), and it only increased in for the EL group in the post-test (*F*_(1, 40)_ = 16.24; *p* = .0002). No significant effect of the Test or the Learning was observed on the selection of the A card (true antecedent; *F*_(1, 40)_ = 1.36; *p* = .25 and *F*_(1, 40)_ = 0.19; *p* = .67, respectively) and the D card (the false antecedent; *F*_(1, 40)_ = 0; *p* = 1 and *F*_(1, 40)_ = 0.76; *p* = .39, respectively).

Moreover, we observed that the participants who received EL changed their card selection between the pre- and the post-tests. They tended to inhibit the selection of the irrelevant matching card (3) in the post-test (Mac Nemar’s χ_(1)_^2^ = 3.13; *p* = .07 for selection → no selection pattern; χ_(1)_^2^ = 0; *p* = 1 for CL group), and most participants activated the selection of the relevant non-matching card (7) in the post-test (Mac Nemar’s χ_(1)_^2^ = 7.69; *p* = .005 for no selection → selection pattern; χ_(1)_^2^ = 0; *p* = 1 for CL group). We observed that more participants gave logical responses after EL than after CL (6 *vs*. 2, respectively), but the difference between the number of erroneous matching responses toward logical responses between the pre- and post-tests in the EL group was not significant (Mac Nemar’s χ_(1)_^2^ = 1.33; *p* = .25 and χ_(1)_^2^ = 0.5; *p* = .48 for CL group).

## Discussion

The question raised in this study was whether the transfer of knowledge acquired with EL proposed by Houdé and Moutier [9] remains effective in identical, but reversed, reasoning tasks that differed in heuristic-inhibiting metacognitive cost. Three major results emerged from our investigation: (i) only EL contributed to shifting the participants’ focus away from the irrelevant matching element named in the conditional rule, (ii) EL permitted the engagement in hypothetical thinking in a portion of the participants, and (iii) CL, as has previously been shown, did not remove reasoners’ biases toward the matching heuristic.

Our results again demonstrate the effect of EL, which removes the focus the participants placed on the matching heuristic. The post-test response distributions were significantly different between the participants who received EL and the participants who received CL. We observed that the majority of the EL group who utilized the matching heuristic in the pre-test gave a different response in the post-test. EL allowed reasoners to inhibit the matching card (3) and select the non-matching card (7), which is the key difficulty in the cards task and the most frequently forgotten part of the logical answer. Nevertheless, contrary to the results obtained in previous studies [9], [12], we did not observed a significant difference in proportions of the biased and logical sub-populations within the EL condition compared to the CL condition in the post-test. EL is limited because it did not cause all of the EL participants to improve. The executive warnings alone were not helpful in inhibiting the tempting matching heuristic generated by System 1 to apply the logical strategy provided by the System 2. We assume that the acquired elementary metacognitive knowledge was “strictly” transferred to discourage matching rather than encourage logic. This underlines the complexity of learning transfer and adds a new piece of evidence to the road toward pedagogy of reasoning.

EL consists of transferring knowledge acquired on a conditional reasoning task to another isomorphic task (in this study, from the RFT to the WST). However, the tasks differ in presentation. A negation presented in pre- and post-tests that supports the learning phase appeared in the RFT but did not appear in the WST. We used these two conditional rules because they elicit a similar heuristic strategy toward the matching bias, which conduct a reasoning error in these two cases and are based on the same analytic strategy. These two conditions are necessary for executive learning, which is used to redirect reasoners’ thinking from error to logic [9]. This factor is an essential feature of our design and does not constitute a confound. If we had chosen to use a different version of the RFT with no negation in the conditional rule, then the matching heuristic strategy would have been easy to falsify for our subjects because the logical inference (*i*.*e*., *the* so-called “*modus tollens*”) did not interfere with matching bias. Additionally, the RFT condition without negation is well known to lead participants to improved performances. Indeed, as the RFT task without negation does not elicit matching-bias strategy, it could not be used as a starting point for a matching-bias inhibition learning. Therefore, both of the used conditional reasoning tasks require the following: 1) inhibition of a heuristic strategy at the attentional level (matching heuristic), and 2) access to the analytic strategy, which allows participants to give the same logical response (i.e., select the true antecedent and the false consequent cards).

Based on the RFT, the participants learned the irrelevance of cases named in the rule. This acquired knowledge became more “strict” when it was supplied as verbal and visuo-spatial executive warnings. We observed that the EL leads to a significant reduction in matching selections on the WST without increasing logical performances. The correct answer to the RFT has the same logical status as the correct answer to the WST, which was presented as the post-test. However, in the case of the WST, the correct answer contains a matched card (A) due to the absence of a negation in the antecedent of the rule. Therefore, reasoning in the post-test task required that the participants understand that it was necessary to only consider one matching card as irrelevant (3) whereas the A card was relevant. Although only EL was effective in permitting the inhibition of the matching card (3) and the activation of the non-matching card (7), some participants included in this learning group applied the previously acquired knowledge to the RFT and considered all cases named in the conditional rule irrelevant. Alternatively, one could argue that EL allows the reasoners to infer that his/her performance on the reasoning task presented in pre-test (the WST) was incorrect. However, in both conditions (EL and CL) the learning phase began based on the participants's response on the RFT, for which the experimenter explicitly explains that it is false. By subtraction, only the logical components remain present in the two learnings. Then, the experimenter explains logically to the two groups why the given response on the RFT is false. Therefore, all participants could infer that his/her performance on the reasoning task presented in pre-test (the WST) was incorrect.

Our results are consistent with the negative priming effect of EL [12], [52]. The participants learned about the WST. The RFT, which was presented in the pre- and post-tests, was modified by introducing a negation in the consequent of the conditional rule (i.e., if there is a red square on the left, then there is not a yellow circle on the right). In this case, the matching heuristic response (activation of all matching cases named in the rule) corresponds to the logical response. In these studies, the participants succeed in the pre-test, and EL had a negative effect on post-test performance. EL created a cognitive conflict in the post-test by discouraging matching rather than by encouraging logical thinking. The knowledge acquired in the learning phase on the WST (half-suppression of the matching elements) led the participants to inhibit the correct answer in the post-test [12], [52]. Dual process theories of reasoning explain the human tendency to violate the normative standards of logic by distinguishing Systems 1 and 2 of thinking [2], [3]. Fast and unconscious processes based on prior knowledge (System 1) may lead to bias in situations that require complex reasoning. In these cases, the analytic system (System 2) must override the responses produced by the heuristic system [4]. Even if it has been shown that reasoners detect the conflict between the outputs of the two systems and are able to monitor System 1 [30]-[32], we argue that in the present study, the knowledge acquired from EL on the RFT prevented the participants from detecting the conflict between the matching heuristic and the analytic strategy in the WST that was presented post-test. Indeed, EL, in the absence of efficient bias detection, might therefore result in a general shutdown of the heuristic route [58] and lead the participants to consider all matching cases, including the relevant A card, as irrelevant. One could argue that our results are because EL contains more information than CL, which is—in part—different from CL and introduced differences in quantity and variety of intervention. It is unlikely given that Osman [28] has observed that many exposures to the same task do not improve performance in the WST (a test-retest procedure without any learning). We observed the same result in our CL condition, which by contrast contains more information than the test-retest condition used by Osman. Therefore, increasing the quality and variety of intervention is not sufficient to improve logical reasoning. Therefore, we believe that an intervention based on cognitive control processes is needed. According to our knowledge, only Osman [28] has conceived a successful tutoring procedure based on identifying and modifying the types of misunderstandings participants had of the WST. This tutoring seems similar to our CL that did not lead to significant improvement in success in our study. However, the specificity of our learning procedure (CL and EL) was not used in the same task in the pre-test and post-tests, and in the learning phase. Indeed, a core feature in education is the capability to transfer acquired knowledge to other tasks and contexts. In the present study, CL was not sufficient to transfer learned knowledge from the RFT to the WST. In contrast, we showed that the transfer relies on executive components that seem necessary to block the heuristic strategy. Our findings add evidence that executive function can be viewed as a core component of learning [53]-[54]. Designing learning that allows the learner to transfer knowledge to new problems is an important challenge for educational psychology in particular [55]. In this context, the effects of EL on selective attention capacities [56], mathematics, and spelling abilities [57] have been successfully tested in school settings. Furthermore, neural data provide evidence implicating inhibitory processes that override the heuristics and open the doors to a neuropedagogy [48]-[49]. It is known that learning is one of the most important factors that results in reorganization of the neural systems involved in cognitive functions [64 58]. Therefore, the idea that influencing brain networks can have implications for education has recently been strongly promoted [59].

Henceforth, determining the contexts that facilitate transfer will not be sufficient. Additional measures should be included in future work. We propose the use of a confidence scale at all steps of the procedure to measure conflict detection between the heuristic and the analytic responses [60]. Moreover, verbal protocols should be used to identify the knowledge that is transferred and how that knowledge is transferred. According to the theory of multiple mechanisms and adaptive shifting, knowledge transfer depends on both the type of knowledge to be transferred and the processing demands of the transfer task [61]. Although the sample size used in deductive reasoning learning studies was classically approximately twenty reasoners per condition [9], [12], [19], future studies could use larger sizes. Finally, it will be interesting to test the contribution of executive processes to overcoming the matching heuristic at the neural level in the WST. Future investigations of EL should explore these important issues.

## Supporting Information

S1 Dataset Complete instructions used in the study and raw values. (DOCX)
