# Supplementary material for: Helping Reasoners Succeed in the Wason Selection Task: When Executive Learning Discourages Heuristic Response but Does Not Necessarily Encourage Logic
Source: PLoS One. 2015 Apr 7;10(4):e0123024. doi: 10.1371/journal.pone.0123024 (PMC4388638; doi:10.1371/journal.pone.0123024)
Supplement: S1 Dataset — (DOCX) [file pone.0123024.s001.docx]

**S1 dataset**

**Complete instructions used in this study**

The learning stages were supported by ‘executive instructions’ or ‘logical instructions’ about the conditional rule falsification task [42]. The former included logical analysis of the task and the trap the task involved. Executive learning (EL) specifically presented the warning elements both verbally—in italics in the following instructions—and visuospatially (see Figure 2B, board a). The latter consisted of presenting participants with a logical (classical) analysis of the task without the inhibition components. Thus, what remained of the learning instructions was the logical explanation of the correct and incorrect answers in normal print. A board is placed on the table facing the participant and the experimenter is sitting next to him. Twelve colored geometrical figures, a double box, and the conditional rule written on a sheet are placed on the board. When the experimenter explains the RFT, he points sequentially to the components of the task.

The experimenter began by saying the following:

“In this problem, the source of the error lies in a habit we all have of concentrating on geometric figures with the shape or color mentioned in the rule [the experimenter points to the red square and the yellow circle and to the places they were mentioned in the rule] and not paying attention to the other figures. *This can have a very misleading effect on us: we think this makes things easier when in fact we’re falling into a trap!* Thus, the goal here is *(1) to not fall into the trap of the two colored figures, the red square and the yellow circle, and (2)* consider all of the colored figures, squares, circles, diamonds in green, red, yellow and blue [the experimenter points to all figures] by imagining if each one can or cannot be placed on the left or the right of the double box drawn on the board [the experimenter points to the double box] to see whether these figures can make the rule false. To help you understand, let’s consider the different answers and eliminate the wrong ones *- the ones that make fall into the trap -* to find the right answer”.

Then, the participant was shown the second part of the experimental materials. During EL a response repertoire was depicted as a box on the board. The inhibition process was visualized with a transparent hatched area on top of the box, and the activation process was depicted as an unhatched circle cut-out from the middle of the hatched area (see Figure 2B, board a). Some answers were represented on small cards that can be slid into the response repertoire in the EL only. These cards were of different colors, depending on whether the answer was correct (i.e., true antecedent-false consequent) or wrong (i.e., total matching false antecedent-true consequent; partial matching false antecedent-false consequent and true antecedent-true consequent). During classical logical learning participants were given another board placed right next to the first (see Figure 2B, board b).

The experimenter said the following:

“On the board you see here, we’re going to put the different answers written on these cards, while clearly separating the wrong answers, which make you fall into the trap, we’ll put them under the hatching, from the right answers. Let’s start with your answer red square on the left and yellow circle on the right [the experimenter takes the corresponding answer card], which means that a red square on the left and a yellow circle on the right proves that the rule is false [the experimenter points to the answer card and to the rule]. When you answer this, you also think it’s easy, and you focus only on the colored figures mentioned in the rule. This is the same trap we talked about above. Thus, to not fall into the trap, slide answer red square-yellow circle under the hatched area [the experimenter does this] because it’s a wrong answer. Let me explain. Putting a red square on the left is a bad choice; the first part of the rule concerns only the colored figures that are not a red square. So, we can place only colored figures that are not a red square on the left, a green circle for example. As a result, the first part of the rule is true (not a red square), and so applicable. But I remind you that you must make the rule totally false. You’ll have to 1) make the rule applicable, that is making the first part of the rule true like we have done and 2) make the second part of the rule false. The second part indicates that we must have a yellow circle on the right. So, putting a yellow circle on the right is a bad choice: we must put any colored figures except a yellow circle on the right, for example a red diamond. As a result, we do not have a red square on the left and a yellow circle on the right. This goes rule, and we have made the rule false.

In conclusion, if you don’t fall into the trap at the beginning thinking it’s easy and concentrating only on the geometric figures mentioned in the rule, the red square and the yellow circle, one of the correct answers is a green circle on the left and a red diamond on the right. So, we put this card in the unhatched circle [the experimenter does this by putting the green circle-red diamond answer card in the unhatched circle]. If I’ve said to you there are actually several possible correct answers. As we have seen, we must put any colored figures, except a red square, on the left and any colored figures, except a yellow circle, on the right. So, for example, placing a green square on the left and a blue circle on the right is also a right answer [the experimenter points to the colored figures]. You must also understand that the rule says “If there is not a red square on the left, then there is a yellow circle on the right” and that it doesn’t say the reverse, if there is a yellow circle on the right, then there is not a red square on the left [the experimenter points the rule]”.

The experimenter continued the learning in the same manner for the two other partial matching biases (false antecedent-false consequent and true antecedent-true consequent) and focused on the warning elements only for the erroneous part of the answer. The learning ended when the participant was capable of producing correct explanations (without prompting from the experimenter) for the following wrong answers: red square-yellow circle; blue diamond-yellow circle, and red square-green diamond.

The experimenter said the following:

“I think that by now you know what you have to do to not fall into the trap and find the right answer. I would like you to re-explain it to me. First with the wrong answer red square on the left and yellow circle on the right”. The experimenter gave the participant the red square and yellow circle answer card and asked him/her to explain using the materials laid out on the two boards. If his/her explanation was correct, she/he went on to blue diamond on the left and yellow circle on the right the wrong answer and then to the red square on the left and green diamond on the right wrong answer. If one of the explanations was incorrect, the experimenter went over the explanation for that answer (red square-yellow circle; blue diamond-yellow circle; red square-green diamond).

To conclude, the experimenter said the following:

“So in this problem, you should not let yourself be dangerously misled by the colored figures mentioned in the rule [the experimenter points to first the two colored figures and then to the places where were mentioned in the rule], which makes you neglect all the other colored figures. You might think this makes things easier, but in fact you fall into a trap! You must also understand that if the rule was “If there is a red square on the left, then there is a yellow circle on the right”, one of the right answers will be to put a red square on the left and a green diamond on the right. In this case, the first part of the rule says that there is a red square on the left, so to make rule applicable by making the first part of the rule true, putting a red square on the left is a good choice. To not fall into the trap of this rule [the experimenter points the new rule placed in another board] just one of the two colored figures, the yellow circle, can have a very misleading effect on us when the other, the red square, is correct.

**Raw values**

| **Age** | **Sex** | **Pre-Test WST** | **Response Category** | **Performance** | **RFT** | **Response Category** | **Performance** | **Learning** | **Post-Test WST** | **Response Category** | **Performance** |
| --- | --- | --- | --- | --- | --- | --- | --- | --- | --- | --- | --- |
| 23,2 | F | FA - FC | no matching | Failure | TA - FC | no matching | Sucess | CL | TA - TC | total matching | Failure |
| 19,8 | F | TC | partial matching | Failure | FA - TC | total matching | Failure | CL | TA | partial matching | Failure |
| 20,8 | F | TA - TC | total matching | Failure | FA - TC | total matching | Failure | CL | TA - TC | total matching | Failure |
| 21 | F | TA | partial matching | Failure | FA - TC | total matching | Failure | CL | TA | partial matching | Failure |
| 19,1 | F | TA | partial matching | Failure | FA - TC | total matching | Failure | CL | TA | partial matching | Failure |
| 20,2 | F | TC | partial matching | Failure | FA - TC | total matching | Failure | CL | TC - FC | no matching | Failure |
| 32,7 | M | TA - TC | total matching | Failure | FA - TC | total matching | Failure | CL | TA - TC | total matching | Failure |
| 19,7 | M | TA - TC | total matching | Failure | FA - TC | total matching | Failure | CL | TA - FC | no matching | Sucess |
| 18,1 | F | TA - TC | total matching | Failure | FA - TC | total matching | Failure | CL | TA | partial matching | Failure |
| 18,9 | F | TA - TC | total matching | Failure | TA - TC | partial matching | Failure | CL | TA - FC | no matching | Sucess |
| 18 | F | TA - TC | total matching | Failure | FA - TC | total matching | Failure | CL | TA - TC | total matching | Failure |
| 17,1 | F | TA - TC | total matching | Failure | FA - TC | total matching | Failure | CL | TA - TC | total matching | Failure |
| 18,4 | F | TA - TC | total matching | Failure | FA - TC | total matching | Failure | CL | TA - TC | total matching | Failure |
| 18,5 | F | TA - TC | total matching | Failure | FA - TC | total matching | Failure | CL | TA - TC | total matching | Failure |
| 18,8 | F | FA - FC | no matching | Failure | FA - TC | total matching | Failure | CL | FA - FC | no matching | Failure |
| 18,6 | F | TA - TC | total matching | Failure | FA - TC | total matching | Failure | CL | TA - FA - TC - FC | no matching | Failure |
| 24,2 | F | FA - FC | no matching | Failure | FA - TC | total matching | Failure | CL | FA - TC - FC | no matching | Failure |
| 18,2 | F | TA - FA - TC - FC | no matching | Failure | FA - TC | total matching | Failure | CL | TA | partial matching | Failure |
| 20,1 | M | FA - FC | no matching | Failure | TA - FC | no matching | Sucess | CL | TA - FA - TC - FC | no matching | Failure |
| 18,6 | F | FA - FC | no matching | Failure | FA - TC | total matching | Failure | CL | TA - TC | total matching | Failure |
| 18,5 | F | TA - TC | total matching | Failure | FA - TC | total matching | Failure | CL | TA - TC | total matching | Failure |
| 20,8 | F | TA - TC | total matching | Failure | TA - TC | no matching | Failure | EL | TA | partial matching | Failure |
| 20,8 | F | TA - TC | total matching | Failure | FA - FC | no matching | Failure | EL | FA - FC | no matching | Failure |
| 20,3 | F | TA | partial matching | Failure | FA - TC | total matching | Failure | EL | TA - FC | no matching | Sucess |
| 20,8 | F | FA - TC | no matching | Failure | FA - TC | total matching | Failure | EL | TA - FC | no matching | Sucess |
| 18,8 | F | FA - FC | no matching | Failure | FA - TC | total matching | Failure | EL | FA - FC | no matching | Failure |
| 20,3 | M | FA - FC | no matching | Failure | FA - TC | total matching | Failure | EL | TA | partial matching | Failure |
| 21,5 | M | TA | partial matching | Failure | FA - TC | total matching | Failure | EL | TA | partial matching | Failure |
| 20,8 | M | TA | partial matching | Failure | TA - TC | no matching | Failure | EL | TA - FC | no matching | Sucess |
| 19,4 | M | TA | partial matching | Failure | FA - TC | total matching | Failure | EL | TA | partial matching | Failure |
| 18,4 | F | TA - FA - TC - FC | no matching | Failure | FA - TC | total matching | Failure | EL | TA -FC | no matching | Sucess |
| 20,2 | F | TA - TC | total matching | Failure | FA - TC | total matching | Failure | EL | FA - FC | no matching | Failure |
| 18,6 | F | FA - FC | no matching | Failure | FA - TC | total matching | Failure | EL | TA - FC | no matching | Sucess |
| 18,6 | F | TA - TC | total matching | Failure | FA - FC | no matching | Failure | EL | TA - TC - FC | no matching | Failure |
| 18,2 | F | TA | partial matching | Failure | FA - TC | total matching | Failure | EL | TA - TC - FC | no matching | Failure |
| 19,9 | F | FA - FC | no matching | Failure | FA - TC | total matching | Failure | EL | FA - FC | no matching | Failure |
| 19,5 | F | TA - TC | total matching | Failure | FA - TC | total matching | Failure | EL | TA | partial matching | Failure |
| 19 | F | TA - TC | total matching | Failure | FA - TC | total matching | Failure | EL | TA - FC | no matching | Sucess |
| 20,8 | F | TC | partial matching | Failure | FA - TC | total matching | Failure | EL | TA - FA - TC - FC | no matching | Failure |
| 22,1 | F | TA - TC | total matching | Failure | TA - FC | no matching | Sucess | EL | TA - FA - TC - FC | no matching | Failure |
| 18,9 | F | TA - TC | total matching | Failure | FA - TC | total matching | Failure | EL | FA - TC - FC | no matching | Failure |
| 18,9 | F | TA - TC | total matching | Failure | FA - TC | total matching | Failure | EL | FA - TC - FC | no matching | Failure |
